# Molecular Insights and Orthopedic Management in Muscular Dystrophies: A Comprehensive Review

**DOI:** 10.3390/ijms27041896

**Published:** 2026-02-16

**Authors:** Jan Lejman, Michał Pytlak, Anna Danielewicz, Erich Rutz, Michał Latalski, Monika Lejman

**Affiliations:** 1 Department of Children’s Neurology, Medical University of Lublin, 20-093 Lublin, Poland; 2 Department of Children’s Neurology, University Children’s Hospital in Lublin, 20-093 Lublin, Poland; michalpytlak88@gmail.com; 3 Children’s Orthopaedic Department, Medical University of Lublin, 20-093 Lublin, Poland; anna.danielewicz@gmail.com; 4 Department of Orthopaedics, The Royal Children’s Hospital, Melbourne 3010, Australia; erich.rutz@rch.org.au; 5 Department of Paediatrics, Bob Dickens Chair, Paediatric Orthopaedic Surgery, The University of Melbourne, Melbourne 3010, Australia; 6 Independent Laboratory of Genetic Diagnostics, Medical University of Lublin, 20-093 Lublin, Poland; monika.lejman@umlub.edu.pl

**Keywords:** muscular dystrophies, muscle degeneration, gene therapy, molecular pathogenesis, orthopedic management, precision medicine

## Abstract

Muscle degeneration is the hallmark of muscular dystrophies—genetically heterogeneous disorders traditionally approached through the lens of molecular pathogenesis or symptomatic management in isolation. Here, we present a deliberately interdisciplinary synthesis that bridges molecular genetics, clinical phenotyping, and evidence-based orthopedic decision-making to address a significant critical gap: the lack of genotype-informed, function-oriented frameworks for musculoskeletal complications. We re-evaluate disease entities—not only by their molecular etiology (e.g., DMD, LMNA, DUX4 dysregulation), but through the prism of orthopedic manifestations as diagnostic gateways and therapeutic milestones. For instance, early rigid spine in *LMNA*-related dystrophy is not merely a sign of contracture, but a red flag demanding cardiac risk stratification before surgical planning, in alignment with current consensus. Similarly, scoliosis management in Duchenne muscular dystrophy is discussed through quantitative decision thresholds (Cobb angle ≥ 20–30°, FVC ≥ 30–35%) derived from long-term outcome studies, rather than general clinical recommendations. Critically, we confront challenges posed by disease-modifying therapies: patients now survive into their 30s and 40s, yet develop novel, therapy-exacerbated orthopedic phenotypes (e.g., steroid-induced osteoporosis, atypical spinal rigidity). Therefore, we argue that *precision orthopedics*—tailored surveillance, genotype-stratified intervention timing (e.g., D4Z4 repeat-guided monitoring in FSHD, and realistic functional goal-setting (e.g., scapular arthrodesis for overhead function)—should become the gold standard of care. For example, desminopathies may show marked phenotypic variability even within the same mutation. Our review thus serves not only as a molecular overview, but as a practical roadmap for neurologists, geneticists, orthopedic surgeons, and rehabilitation specialists seeking to translate genomic insights into durable functional outcomes.

## 1. Introduction

Neuromuscular diseases are a highly heterogeneous group of disorders characterized by damage to structures vital for nerve impulse transmission and muscle contraction. Within this broad category, muscle degeneration is a core pathological process underlying many of these conditions, leading to progressive muscle weakness, atrophy, significant motor disability, and a reduced quality-of-life [1,2].

Among these, muscular dystrophies are a significant subgroup of progressive, genetically determined diseases characterized by primary skeletal muscle weakness and atrophy. These disorders are most commonly inherited in an X-linked pattern (as in Duchenne muscular dystrophy—DMD), an autosomal recessive pattern (e.g., some forms of limb-girdle muscular dystrophies—LGMD), or an autosomal dominant pattern (e.g., Emery–Dreifuss muscular dystrophy) [3]. Genetic defects lead to damage to myocytes, which are progressively replaced by connective and adipose tissue [4].

In recent decades, advancements in molecular biology and clinical genetics have provided deep insights into the complex mechanisms underlying muscle cell degeneration. Using next-generation sequencing (NGS) techniques, hundreds of mutations have been identified in genes encoding both structural (such as DMD, LMNA, CAPN3, COL6A1, RYR1) and enzymatic proteins [5]. Dysfunction of these proteins triggers a cascade of pathological processes at the cellular level, including sarcolemmal instability, disrupted intracellular signaling, chronic inflammation, and ultimately, progressive muscle atrophy [6].

Understanding these molecular bases has critical clinical implications. It not only facilitates more accurate diagnosis and prognosis but, crucially, underpins the development of targeted therapies. Notably, success and experience with gene therapy in treating spinal muscular atrophy (SMA), such as the approval of innovative drugs, have significantly accelerated the research and development of similar therapeutic strategies for different types of muscular dystrophies. These advancements in SMA have opened new perspectives for gene therapies utilizing AAV vectors, exon-skipping therapies, and the pharmacological modulation of cellular pathways in muscle diseases [7].

Early diagnosis of muscular dystrophies is therefore critical. Prompt recognition, often facilitated by modern genetic methods, is crucial for optimizing interdisciplinary medical care, enabling the rapid implementation of rehabilitative interventions, planning comprehensive symptomatic and pharmacological treatments, and monitoring disease progression and complications. This allows the early initiation of appropriate support, which can significantly slow symptom progression, improve the quality-of-life of patients and their families, and prepare them for future health challenges [8].

While advances in genetic diagnostics and gene therapy have revolutionized the biological understanding of muscular dystrophies, their clinical translation remains fragmented. Molecular discoveries rarely inform clinical decisions, such as when to operate on scoliosis (cf. FVC thresholds and survival benefit [9,10,11]), how to weigh cardiac risk against contracture release in LMNA carriers [12,13,14], or what functional gains justify high-morbidity procedures like scapulothoracic arthrodesis in FSHD [15,16]. Consequently, orthopedic care—though pivotal for quality-of-life—is often relegated to “supportive” status, lacking integration with genotype-specific natural history data.

This review challenges that dichotomy. Our primary aim is not merely to catalog disease mechanisms, but to build a clinically actionable bridge between genotype and orthopedic phenotype and early management. We propose that musculoskeletal manifestations are not just complications—they are early diagnostic clues, progression biomarkers, and therapeutic decision points, all of which demand interpretation in the context of the underlying molecular pathology.

To this end, we reframe pathomechanisms around their functional orthopedic consequences (e.g., nuclear fragility in LMNA → rigid spine → altered surgical risk [17,18,19]), revise management sections with expert-level criteria (e.g., FVC thresholds [9,11], D4Z4 repeat stratification [20]), and critically analyze gaps exposed by modern therapies—including the “long-term-survivor paradox” (prolonged life with novel comorbidities [21,22]), and also conclude with a forward-looking framework for genotype-stratified orthopedic care.

By unifying neurology, genetics, and orthopedics, we aim to provide more than a review—we offer a foundation for precision functional medicine in muscular dystrophies.

### Search Strategy and Selection Criteria

For this comprehensive review, we conducted a search across PubMed, Scopus, and Google Scholar databases for articles published between January 2000 and May 2024. The search terms included “muscular dystrophy”, “orthopaedic management”, “molecular pathogenesis”, “genotype-phenotype correlation”, “spinal deformity”, and “contractures”. We prioritized peer-reviewed original research articles, longitudinal natural history studies, and international clinical guidelines. Only English-language full-text articles were included. Studies with very small cohorts (n < 5) were excluded unless they provided unique molecular insights into rare MD subtypes.

## 2. Common Pathomechanisms Across Muscular Dystrophies

Muscular dystrophies (MDs) are a genetically heterogeneous group of disorders, yet they share a common final pathway: the progressive structural and functional failure of skeletal muscle, culminating in weakness, contractures, and spinal deformity.

While the causative genes differ, they converge on a common downstream effect: an increased biomechanical vulnerability of the muscle fibers. Importantly, many of these molecular lesions directly compromise the muscle’s ability to withstand mechanical load. This renders patients susceptible not only to atrophy but also to specific orthopedic phenotypes that often precede overt weakness and can guide early diagnosis [18,23,24].

**Figure 1:**
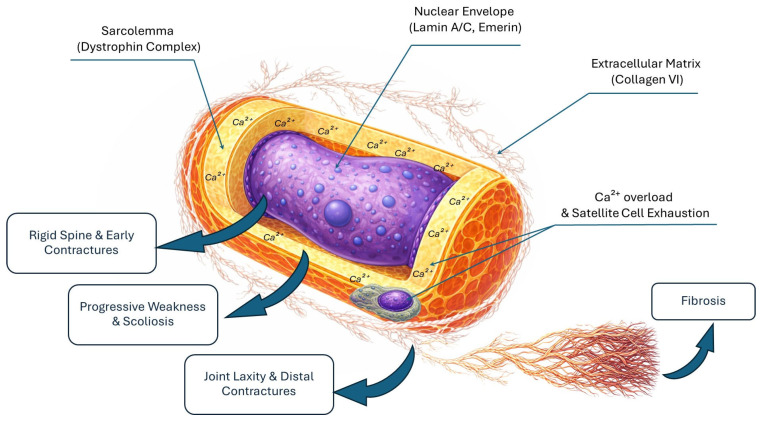
Integrated molecular–orthopedic landscape of the cylindrical muscle fiber. The schematic illustrates the anatomical localization of key proteins and their respective clinical phenotypes through an “Inside-Out” framework. (1) The nucleus (center): Mutations in nuclear envelope proteins (lamin A/C, emerin) drive early axial stiffness and rigid spine. (2) The sarcolemma (boundary): Deficiency of the dystrophin complex leads to chronic calcium (Ca^2+^) influx into the cytoplasm, resulting in progressive limb weakness and scoliosis. (3) The extracellular matrix (exterior): Defects in collagen VI disrupt force transmission, causing the paradox of joint laxity and distal contractures. Satellite cells, located in their niche between the sarcolemma and matrix, undergo exhaustion and loss of polarity, leading to failed regeneration and progressive fibrosis. This irreversible replacement of contractile tissue leads to severe, end-stage clinical contractures.

Below, we reframe these classical pathomechanisms not merely as cellular events, but as drivers of functional decline whose timing and severity dictate the optimal windows for early and accurate orthopedic intervention (Figure 1).

### 2.1. Nuclear Instability and Mechanotransduction Failure

Lamin A/C and emerin transmit mechanical signals from the cytoskeleton to the nucleus, regulating gene expression in response to load [25,26]. LMNA or EMD mutations disrupt this “mechanosensing,” leading to aberrant gene activation (e.g., TGF-β, p38 MAPK) even at low mechanical stress [18,27]. This explains why EDMD patients develop contractures despite minimal weakness—and why these contractures are disproportionately severe in load-bearing postural muscles [17,28].

Orthopedic correlate: The “triad” of EDMD—elbow, Achilles, and cervical contractures—is not random; it reflects the anatomic hierarchy of tonic postural demand. This pattern is so specific that it should trigger LMNA/EMD testing before comprehensive NGS panels [12,17].

### 2.2. Mechanical Failure of the Sarcolemma and Force-Transmission System

The dystrophin–glycoprotein complex (DGC) acts as a critical shock absorber during muscle contraction, anchoring the cytoskeleton to the extracellular matrix (ECM). In DMD and sarcoglycanopathies, the absence or dysfunction of dystrophin or sarcoglycans disrupts this mechanical buffering, leading to sarcolemmal microtears with each contraction cycle [24,29,30]. This results in uncontrolled Ca^2+^ influx, protease activation, and fiber necrosis. Importantly, it also causes asymmetric fiber loss and regional fatty replacement, which destabilizes joint mechanics long before widespread weakness manifests [31,32].

Orthopedic correlate: In DMD, the earliest biomechanical failure is often at the ankle–plantarflexor interface, where eccentric loading during gait promotes preferential gastrocnemius–soleus degeneration → equinus contracture → altered gait kinematics → hip/knee flexion contractures [20,33]. In LMNA-related dystrophies, the same principle applies to postural muscles: paraspinal and posterior cervical fibers, which are constantly under a low-level tonic load, degenerate early, leading to the hallmark rigid spine and cervical hyperlordosis [17,18,19].

### 2.3. Impaired Force Transmission via the Extracellular Matrix

Collagen VI and laminin-α2 form a compliant “molecular mesh” linking myofibers to the basement membrane. Mutations in COL6A or LAMA2 disrupt this elasticity, rendering fibers highly sensitive to mechanical strain [34,35]. In the Bethlem/Ullrich spectrum, this manifests as early-onset, severe joint laxity followed by rapid contracture progression—a biomechanical paradox reflecting a loss of tissue resilience [34].

Orthopedic correlate: The combination of ligamentous laxity and muscle weakness predisposes foot deformities (pes planovalgus, hindfoot instability) and hip subluxation, requiring dynamic orthoses (e.g., supramalleolar orthoses) rather than rigid AFOs [36,37].

### 2.4. Calcium Dysregulation and Oxidative Stress: Accelerators of Tissue Stiffness

Beyond primary structural defects, calcium dysregulation and oxidative stress represent universal downstream pathways across the spectrum of muscular dystrophies.

Chronic Ca^2+^ overload, whether resulting from membrane rupture (DMD), ryanodine receptor leakage (RYR1), or impaired extrusion, activates calpains and caspases, driving proteolysis and mitochondrial dysfunction [38,39]. This cascade promotes fibrosis and fatty infiltration—processes that not only replace contractile tissue but also increase passive tissue stiffness [23,40]. Oxidative stress further cross-links collagen, reducing tissue elasticity [38].

Orthopedic correlate: The resultant non-elastic contractures (elbows in EDMD, Achilles in LGMD) resist standard stretching and orthoses. This makes the timing of intervention crucial: once fibrosis dominates (e.g., when passive ROM loss exceeds 5°), only surgical release restores function [36,41]. Significantly, glucocorticoid therapy in DMD delays this fibrotic transition but exacerbates osteoporosis, creating a new orthopedic trade-off [21,42].

### 2.5. Failed Regeneration: The Role of Satellite Cell Exhaustion

Chronic muscle damage in MDs eventually leads to the exhaustion of the regenerative capacity of satellite cells (SCs). Recent evidence suggests that in DMD and other “satellite cellopathies,” the pool of SCs is not only depleted but also functionally impaired due to a loss of cell polarity and asymmetric division [43,44]. This impaired repair mechanism accelerates fatty infiltration [45,46] and the progressive replacement of contractile tissue with dense fibrous connective tissue [10,20].

Orthopedic correlate: “Failed regeneration” and the subsequent fibro-fatty transformation are key drivers of progressive muscle shortening and permanent loss of joint mobility, making early intervention and pharmacological support of muscle quality crucial [10,20].

### 2.6. The Vicious Cycle: From Molecular Lesion to Functional Collapse

These mechanisms feed into a self-reinforcing loop: genetic defect → mechanical fragility → microinjury → Ca^2+^/ROS → inflammation/fibrosis → reduced elasticity → altered joint loading → accelerated degeneration → deformity.

This cascade explains why orthopedic milestones (e.g., loss of ambulation, scoliosis onset) are more reproducible than serum CK or even muscle strength—they reflect the integrated biomechanical failure of the system [9,33].

Clinical implication: Monitoring should shift from isolated strength testing to functional biomechanics, including 3D gait analysis (3-DGA), 3D spinal tracking, and passive ROM trends—ideally stratified by genotype (e.g., DMD: monitor FVC and Cobb; LMNA: monitor cervical ROM and ECG) [9,11,13,21].

## 3. Genetic, Clinical, and Pathophysiological Profiles of Specific Muscular Dystrophies

While shared pathomechanisms (Section 2) define the final common pathway of muscle degeneration, the clinical trajectory, orthopedic complications, and therapeutic windows are fundamentally shaped by genotype. Below, we reframe classical disease entities not as isolated diagnostic labels, but as dynamic genotypic–phenotypic continua, where early recognition of orthopedic patterns can trigger timely genetic testing—and where molecular diagnosis dictates not only prognosis, but the timing and type of orthopedic intervention. This new way of understanding will enhance better management in an interdisciplinary approach.

### 3.1. Duchenne and Becker Muscular Dystrophies

Duchenne (DMD) and Becker (BMD) muscular dystrophies are X-linked disorders caused by mutations in the *DMD* gene (Xp21), which encodes dystrophin—an essential mechanical linker between the actin cytoskeleton and the dystrophin–glycoprotein complex (DGC) [47,48]. Absence (results in DMD) or truncation (results in BMD) of dystrophin severely compromises membrane buffering during contraction, leading to sarcolemmal microtears, Ca^2+^ overload, and progressive necrosis [24,31]. This chronic influx of calcium activates intracellular proteases, such as calpains, which further degrade myofibrillar proteins and trigger an inflammatory cascade. Over time, the exhausted regenerative capacity of the muscle leads to progressive replacement of contractile fibers with dense fibrous connective tissue and adipose infiltration. From an orthopedic perspective, this fibrotic transformation is the primary molecular driver of progressive muscle shortening, leading to fixed joint contractures and severe spinal deformities that often necessitate surgical intervention [5,6,22]. Clinically, DMD manifests before age 5 with delayed motor milestones, Gowers’ sign, calf pseudrohypertophy, and elevated CK (>10,000 U/L) [44,49]. Loss of ambulation typically occurs between 10 and 12 years [50,51,52]. This is a critical orthopedic inflection point: non-ambulation triggers rapid progression of neuromuscular scoliosis (mean Cobb progression: 10–15°/year [12]) and multilevel contractures (ankle, knee, hip, elbow) [20,33]. Importantly, scoliosis management must be timed to pulmonary status: spinal fusion is recommended only when forced vital capacity (FVC) remains ≥30–35% of its predicted value—a threshold linked to reduced perioperative mortality and more prolonged survival (median gain: 3.2 years [11]). Delaying surgery until FVC < 30% significantly increases morbidity; yet performing it too early, before curve progression is evident, exposes the patient to unnecessary risk.

Cardiac involvement is near-universal in DMD and typically manifests as dilated cardiomyopathy after age 10 [33]. Glucocorticoids (prednisone/deflazacort), while prolonging ambulation as a positive effect, exacerbate bone fragility and weight gain—creating a therapeutic trade-off: improved motor function at the cost of increased fracture risk and surgical complexity [21,42]. Multidisciplinary care (including cardiology, pulmonology, orthopedics, and rehabilitation) has extended median survival from ~17 years (1990s) to ~30 years [21], turning DMD into a chronic condition with novel, therapy-induced orthopedic phenotypes.

BMD, caused by in-frame *DMD* mutations, produces partially functional dystrophin and a milder, more heterogeneous course and clinical presentation [53]. Ambulation may be preserved into adulthood, yet cardiomyopathy remains a leading cause of death—sometimes preceding significant weakness [54]. This highlights the principle that cardiac surveillance should not be deferred until motor decline occurs.

Therapeutically, DMD is now the prototype for precision gene therapy, with microdystrophin AAV delivery (e.g., Elevidys [55]), exon skipping (eteplirsen, golodirsen [56,57,58,59]), and novel anti-inflammatory agents (vamorolone, givinostat [60,61]) reshaping the natural history. Yet their long-term impact on orthopedic milestones—e.g., age at loss of ambulation, scoliosis onset, need for spinal fusion—remains unknown [21]. Will gene therapy delay—but not eliminate—the need for orthopedic intervention? Current guidelines [10,22] do not yet address this gap.

### 3.2. Facioscapulohumeral Muscular Dystrophy (FSHD)

FSHD (prevalence 1:8000–20,000) is an autosomal-dominant, slowly progressive dystrophy driven not by protein loss, but by toxic gain-of-function: aberrant expression of the embryonic transcription factor DUX4 in skeletal muscle [62,63]. In 95% of cases (FSHD1), this results from contraction of the D4Z4 macrosatellite repeat array (1–10 units vs. 11–100 in controls) on a permissive 4qA haplotype; in FSHD2, normal repeat numbers coexist with mutations in chromatin modifiers (e.g., *SMCHD1*) [64].

DUX4 activation induces a cascade of pro-apoptotic, pro-inflammatory, and oxidative stress genes, causing focal myofiber degeneration and fatty replacement in a highly stereotyped pattern: face → scapular stabilizers → abdominal/trunk → distal lower limbs [62,63]. Weakness is typically asymmetric, with relative sparing of forearm and hand muscles—a key differential from LGMD.

From an orthopedic standpoint, the hallmark is scapular winging due to serratus anterior and trapezius weakness [65]. This is not merely a sign—it is a functional bottleneck: impaired arm elevation above 90° limits self-care (feeding, grooming, transfers) and accelerates shoulder pain and fatigue [16]. In selected patients, scapulothoracic arthrodesis can restore overhead function, but outcomes are highly dependent on preoperative residual serratus activity and trunk control [15,16]. This surgery improves a specific functional deficit but does not alter disease progression, and it carries significant risks (rib fracture, pneumothorax, chronic pain). Thus, realistic goal-setting (e.g., “enable self-toileting” rather than “cure FSHD”) is essential.

Lumbar hyperlordosis, caused by abdominal weakness and iliopsoas dominance, is another disabling feature—often accompanied by chronic low-back pain [65,66]. Unlike DMD, respiratory and cardiac involvement are rare, and life expectancy is generally near normal. Yet quality-of-life is significantly impaired by pain, fatigue, and functional loss—necessitating proactive pain management and ergonomic adaptation [66,67].

Importantly, D4Z4 repeat number stratifies risk: patients with 1–3 repeats often present with infantile-onset FSHD—severe, rapidly progressive, with facial weakness before age five and hearing/retinal involvement [64,68]. This justifies genotype-guided surveillance: patients with very short arrays warrant earlier ophthalmologic and audiologic screening, as well as more intensive functional monitoring.

### 3.3. Limb-Girdle Muscular Dystrophies (LGMD)

LGMD encompasses more than 30 genetically distinct disorders unified by predominant weakness of the pelvic and shoulder girdles, but with divergent natural histories, cardiac risks, and orthopedic phenotypes [69]. Classification into recessive (LGMDR) and dominant (LGMDD) forms reflects inheritance patterns, but clinical stratification must be genotype-driven.

Due to significant clinical and genetic heterogeneity, the key LGMD subtypes, their clinical features, and their implications for orthopedic management are summarized in Table 1.

A unifying challenge across LGMDs is the lack of disease-modifying therapies—management remains supportive. Yet emerging gene therapies (e.g., AAV-mediated SGCB delivery [78]) offer hope. As with DMD, their success will be measured not only by biomarkers but also by delayed orthopedic milestones—e.g., preserved ambulation at age 18 and the absence of scoliosis requiring fusion.

### 3.4. Emery–Dreifuss Muscular Dystrophy (EDMD)

EDMD clearly illustrates the integration of genotype, orthopedic presentation, and cardiac risk. It is defined by the triad of: (1) early, disproportionate contractures (elbows, Achilles, posterior cervical muscles), (2) slow progressive humeroperoneal weakness, and, (3) life-threatening cardiomyopathy with conduction defects [12,17].

While X-linked forms involve emerin (EMD), the majority of cases are autosomal-dominant due to LMNA mutations (lamin A/C) [14,79]. Emerin and lamins maintain nuclear envelope integrity and mechanotransduction—linking cytoskeletal strain to nuclear gene regulation [25,27]. Their dysfunction renders muscle nuclei fragile during contraction and disrupts stress-response signaling (e.g., p38 MAPK [27]), leading to fibrosis, fat replacement, and the disproportionate involvement of tonic postural muscles.

The pattern of contractures is diagnostically pathognomonic, characterized by elbow flexion contractures limiting hand-to-mouth function, Achilles shortening hindering the stance phase, and posterior cervical muscle fibrosis causing a rigid spine and cervical hyperlordosis [17,19]. This triad often appears before significant weakness—making it a red flag for EDMD in any child with “unexplained contractures” [17,28].

In LMNA-related EDMD, orthopedic disability is rarely the cause of death. Instead, sudden cardiac death may occur in subjects with minimal motor impairment, with a 5-year risk of malignant arrhythmia exceeding 40% [12,13]. Thus, any planned orthopedic procedure (e.g., contracture release, spinal fusion) requires prior cardiology clearance: prolonged Holter monitoring, signal-averaged ECG, electrophysiological study (EPS) in high-risk cases, and prophylactic ICD placement—even in asymptomatic carriers [12,13]. In practice, this shifts the care pathway, with genetic and cardiac risk stratification preceding orthopedic consultation, not vice versa.

Surgical management of a rigid spine is high-risk; staged anterior–posterior fusion may be indicated, but instrumentation can fail due to osteopenic bone. In patients with limited life expectancy due to cardiac disease, non-fusion stabilization (e.g., modular TLSO, growing rods in adolescents) may be preferable [19].

EDMD exemplifies why muscular dystrophies must be managed as systemic disorders: the orthopedic surgeon, cardiologist, and geneticist must speak the same language and share a common risk assessment.

## 4. Orthopedic Care and Physiotherapy in Muscular Dystrophies

Orthopedic and rehabilitative care are essential pillars of management in muscular dystrophies, directly influencing functional independence, caregiver burden, and quality-of-life [80]. While disease-modifying therapies are emerging, their clinical impact will be judged not only by molecular endpoints but also by delayed orthopedic milestones—for instance, preserved ambulation beyond age 12 in DMD, the absence of progressive scoliosis requiring fusion, or the retained ability to elevate the arm above shoulder height in FSHD. Therefore, orthopedic interventions must be closely integrated with genetic diagnosis and disease trajectory.

### 4.1. Importance of Early Orthopedic Intervention

Early and systematic surveillance allows timely intervention before irreversible musculoskeletal deformities develop. In DMD, ankle equinus contracture often precedes loss of ambulation by 2–3 years and serves as a biomechanical predictor of gait breakdown [20]. As plantarflexion contracture exceeds 15°, compensatory hip and knee flexion rapidly develops, resulting in gait deterioration, increased energy expenditure, and accelerated fatigue [41]. Night-time ankle–foot orthoses (AFOs), which maintain the ankle at 90°, have been shown to delay contracture progression by 1.5–2 years and prolong ambulation [37,81]. In contrast, standing programs using knee–ankle–foot orthoses (KAFOs) remain valuable in selected cases for improving hip extension and bone loading. However, their use has declined with the advent of effective steroid regimens [81].

In Emery–Dreifuss muscular dystrophy (EDMD), where contractures appear early and disproportionately affect the elbows, Achilles tendons, and posterior cervical muscles, early orthotic intervention should be dynamic (e.g., spring-loaded elbow hinges) rather than rigid, to preserve functional arcs without overstressing fragile muscle fibers [17]. For collagen VI-related dystrophies (Bethlem/Ullrich), where ligamentous laxity coexists with weakness, rigid AFOs may exacerbate joint instability; instead, supramalleolar orthoses (SMOs) provide subtalar control while preserving ankle motion [36]. Importantly, a wait-and-see approach carries high risk—serial goniometry, not subjective impression, should guide escalation of intervention, with a decline of more than 5° in passive dorsiflexion over 6 months warranting orthotic or surgical reassessment [20].

### 4.2. Prevention of Contractures and Mobility Support

Contractures result from the interplay of muscle shortening, fatty–fibrotic replacement, and immobilization in shortened positions [20]. Stretching protocols should be slow and sustained (≥30 seconds) and performed after warming (e.g., cycling). In contrast, ballistic or eccentric loading—although beneficial in healthy muscle—is contraindicated in dystrophinopathies and dysferlinopathies due to heightened sarcolemmal fragility and an increased risk of rhabdomyolysis [73,82].

In facioscapulohumeral muscular dystrophy (FSHD), scapular winging due to weakness in the serratus anterior and trapezius muscles is not merely a clinical sign, but a functional bottleneck that limits arm elevation and contributes to chronic shoulder pain and fatigue [16,65]. While corsets and shoulder harnesses offer symptomatic support, scapulothoracic arthrodesis remains the only intervention proven to restore active elevation beyond 90°, thereby enabling self-care tasks such as feeding and grooming [15,16]. However, realistic goal-setting is essential—surgery does not halt disease progression, and outcomes are highly dependent on preoperative residual serratus activity (confirmed via dynamic ultrasound or EMG) and preserved trunk control [15,16]. The decision must balance the functional gain against the procedural risks, including rib fractures, pneumothorax, and chronic pain.

In EDMD, surgical release of contractures—though sometimes necessary—must be preceded by comprehensive cardiac clearance. Even in patients with minimal motor impairment, LMNA mutations confer a >40% five-year risk of malignant arrhythmia or sudden cardiac death [12,13], making prophylactic implantable cardioverter–defibrillator (ICD) placement a prerequisite for elective surgery in most cases [12].

### 4.3. Surgical and Conservative Management of Scoliosis and Spinal Deformities

Progressive neuromuscular scoliosis is among the most disabling orthopedic complications in non-ambulatory patients with muscular dystrophies, most aggressively in DMD but also in sarcoglycanopathies, LMNA-related dystrophies, and collagen VI disorders [9,19,33]. The decision for spinal fusion is not dictated solely by the Cobb angle, but by an integrated assessment of pulmonary, nutritional, and cardiac status.

In DMD, scoliosis typically initiates 6–24 months after loss of ambulation (median age 11 years) and progresses at a mean rate of 10–15° per year [9]. Current consensus, based on long-term outcome studies and ACTION MD recommendations [9,11,22], supports prophylactic posterior spinal fusion to the pelvis when the Cobb angle exceeds 20–30° and demonstrates progression of more than 10° over 6 months. This is contingent upon three factors: (1) Forced vital capacity (FVC) must remain at or above 30–35% of its predicted value (ideally >40% for optimal perioperative safety),(2). Nutritional status must be preserved (BMI > 15th percentile) and (3). Cardiac function must be stable (left ventricular ejection fraction (LVEF) > 45% in the absence of uncontrolled arrhythmias) [9,11,22].

All-pedicle-screw instrumentation has significantly reduced pseudarthrosis rates and postoperative curve progression [9]. Furthermore, early fusion—performed before FVC declines below 30%—is associated with a median survival gain of 3.2 years, improved sitting tolerance, and reduced caregiver burden [11].

In LMNA-related dystrophies (EDMD or LGMDD1), spinal deformity is often less angular but accompanied by early rigid cervical hyperlordosis and thoracolumbar kyphosis, necessitating complex, staged reconstructions [17,19]. Here, the orthopedic–cardiac risk profile dominates clinical decision-making: patients may exhibit minimal weakness yet carry an exceptionally high arrhythmic risk [12,13]. Consequently, any planned spinal procedure requires prior cardiology evaluation, including prolonged Holter monitoring, signal-averaged electrocardiography, and, in most cases, prophylactic ICD implantation—even in asymptomatic carriers [12,13]. In patients with limited life expectancy due to cardiac disease, non-fusion stabilization (e.g., modular thoracolumbosacral orthoses or magnetically controlled growing rods in adolescents) may be preferable to definitive fusion [19].

In Bethlem and Ullrich myopathies, severe early-onset scoliosis may necessitate growth-friendly instrumentation, though wound-healing complications and skin fragility markedly increase surgical risk [34]. Collectively, these examples underscore that genotype-informed orthopedic planning—not diagnosis-based protocols alone—is now the standard of care.

### 4.4. Rehabilitation and Adapted Physical Activity

Physical rehabilitation remains the cornerstone of non-pharmacological management across all muscular dystrophies. Contemporary evidence indicates that moderate and individually tailored physical activity does not accelerate muscle degeneration, but rather improves endurance, functional capacity, and overall well-being [83]. Low- to moderate-intensity aerobic exercise—such as stationary cycling, swimming, or brisk walking—is generally safe and recommended for maintaining cardiopulmonary fitness and delaying the progression of weakness [83]. However, the type and intensity of exercise must be genotype-informed: eccentric and high-intensity resistance training are contraindicated in dystrophinopathies, dysferlinopathies, and RYR1-related disorders due to the heightened risk of sarcolemmal damage and rhabdomyolysis [82,84].

In clinical practice, strength training may be cautiously introduced in selected patients, but only under close supervision and provided that pain, excessive fatigue, or elevated creatine kinase levels do not occur [82]. Emerging assistive technologies—including body weight-supported treadmill training and robotic exoskeletons such as the Hybrid Assistive Limb (HAL) system—enable safer ambulation practice with partial mechanical support, improving coordination and motivation in patients with limb-girdle or Duchenne muscular dystrophy [85]. Concurrently, occupational therapy and environmental adaptations—such as ergonomic handles, lifting systems, or customized seating—significantly enhance independence and reduce fall risk [86].

Pain management in an interdisciplinary team, particularly in facioscapulohumeral muscular dystrophy where chronic shoulder and lumbar discomfort is prevalent, requires a comprehensive strategy combining physical modalities, posture correction, pharmacological interventions, and psychosocial support [66]. Importantly, regular participation in adapted physical activity contributes not only to physical function but also to improved mood, reduced anxiety and depression, and greater social engagement—factors strongly associated with quality-of-life [66]. Therefore, rehabilitation goals should evolve dynamically with disease progression—from preserving ambulation in early stages, to optimizing seated function and independence in non-ambulatory phases, and ultimately prioritizing comfort and dignity in advanced disease.

## 5. Therapeutic Perspectives in Muscular Dystrophy Treatment

The therapeutic landscape for muscular dystrophies has shifted significantly—from purely symptomatic, multidisciplinary management toward mechanism-based, genotype-targeted interventions. While glucocorticoids, cardioprotective agents, and orthopedic care remain foundational, emerging strategies now aim to correct or compensate for the primary genetic defect, offering hope for disease modification rather than mere palliation.

In Duchenne muscular dystrophy (DMD), glucocorticoids such as prednisone, prednisolone, and deflazacort constitute first-line therapy, prolonging ambulation by 2–3 years and delaying respiratory and cardiac decline; however, their long-term use is associated with significant adverse effects, including weight gain, growth retardation, osteoporosis, and behavioral disturbances [42]. Concurrent cardioprotective strategies—including ACE inhibitors, beta-blockers, and mineralocorticoid antagonists—are essential, given the near-universal development of dilated cardiomyopathy, and have contributed substantially to extending median survival into the fourth decade [22].

Recent advances have enabled the development of precision genetic therapies. The FDA-approved microdystrophin gene therapy Elevidys™ (SRP-9001), delivered via AAVrh74 vector, provides functional dystrophin expression in ambulant DMD boys [55]. Antisense oligonucleotide-mediated exon skipping (eteplirsen [56], golodirsen [57], viltolarsen [58], casimersen [59]) allows restoration of the reading frame for amenable DMD mutations, yielding truncated yet partially functional dystrophin. Novel pharmacological agents—such as the histone deacetylase inhibitor givinostat [60], the dissociative glucocorticoid vamorolone [61], and myostatin inhibitors—further expand the therapeutic options, though challenges remain regarding long-term efficacy, immune responses to AAV, and accessibility [42].

In facioscapulohumeral muscular dystrophy (FSHD), therapeutic development focuses on silencing the toxic gain-of-function driver DUX4. A lipid-conjugated siRNA targeting DUX4 has achieved over 50% knockdown of DUX4-regulated transcripts in human xenograft models, supporting its potential as a muscle-directed therapy [87]. In contrast, small-molecule inhibitors like losmapimod failed to demonstrate clinically meaningful functional benefit in the phase 2b ReDUX4 trial, highlighting the complexity of translating molecular suppression into functional improvement [88]. Symptomatic management remains central, with increasing emphasis on chronic pain control—where experimental cannabinoid-based interventions show promise in refractory cases [89].

For Emery–Dreifuss muscular dystrophy (EDMD), current strategies target downstream consequences of LMNA mutations. Preclinical and early clinical data suggest cardioprotective potential for p38α MAPK inhibition and NAD^+^ supplementation (e.g., nicotinamide riboside), reflecting the centrality of cardiac risk in disease management [27,90,91]. AAV-mediated LMNA gene replacement remains under investigation, but faces challenges related to nuclear envelope targeting and dominant-negative effects.

In limb-girdle muscular dystrophies (LGMD), gene therapy trials are rapidly advancing. In Becker muscular dystrophy—a phenocopy of milder DMD—AAV-follistatin (rAAV1.CMV.huFollistatin344) improved muscle strength and functional performance in phase 1/2a studies [92], supporting potential translation to recessive LGMD subtypes such as dysferlinopathy or sarcoglycanopathy. Similarly, AAV-mediated SGCB delivery has shown dose-dependent rescue of muscle pathology in LGMD R4 mouse models [93].

Yet, despite these breakthroughs, the real-world integration of genetic therapies into clinical practice still faces significant unresolved challenges—particularly at the intersection of molecular response and functional orthopedic outcomes.

### Unanswered Questions and Future Directions

The success of gene therapies has transformed muscular dystrophies from uniformly fatal to chronic, manageable conditions—but this achievement has unveiled novel complexities that require multidisciplinary and interdisciplinary planning. Three major gaps stand out.

Firstly, the long-term-survivor paradox: patients now survive into their 30s and 40s [21], yet develop therapy-exacerbated phenotypes: glucocorticoid-induced osteoporosis in microdystrophin recipients, atypical spinal rigidity in collagen VI disorders, or obesity-related joint overload in previously non-ambulant individuals. There is no consensus on the guidelines for managing secondary deformities—such as adjacent segment disease after spinal fusion, or heterotopic ossification post-orthopedic release—in this new population. Rehabilitation protocols, too, remain empirically derived, lacking evidence for adults with decades of disuse atrophy superimposed on primary myopathy.

Secondly, the evidence gap between trial efficacy and real-world impact: therapies are approved based on short-term biomarkers (e.g., dystrophin expression, 6MWT), but their effect on orthopedic milestones—age at loss of ambulation, requirement for scoliosis surgery, need for upper-limb arthrodesis—remains poorly quantified [21]. Will microdystrophin delay spinal fusion by 2 years? Five? Will it change the indication for surgery—from prophylactic (FVC ≥ 35%) to purely symptomatic? Without long-term, functionally anchored registries, clinicians lack the data needed to counsel families or time interventions.

Thirdly, the lack of genotype-stratified orthopedic care pathways. While genetic diagnosis is now routine, orthopedic surveillance intensity remains largely uniform. Yet emerging data suggest that risk is highly genotype-specific: LMNA carriers require quarterly cardiac–orthopedic co-assessment from diagnosis [12,13]; DMD patients on steroids need biannual bone density and spine X-ray monitoring from age eight [21,81]; whereas FSHD patients with >6 D4Z4 repeats may be monitored annually, unless symptomatic [64]. We propose that future guidelines adopt a precision orthopedics framework—one where surveillance frequency, imaging protocols, and surgical thresholds are explicitly tied to molecular diagnosis, not just clinical stage.

Ultimately, the next decade’s focus should shift from disease modification to functional optimization. A successful outcome in 2030 should not be defined solely by dystrophin restoration, but by durable independence: self-toiletting at age 25, wheelchair-to-bed transfer at age 35, or pain-free shoulder function in FSHD. Achieving this demands deeper integration of neurology, genetics, and orthopedics—not as parallel tracks, but as a single, unified framework for care.

## 6. Summary

Muscular dystrophies are genetically heterogeneous disorders unified by progressive structural and functional failure of skeletal muscle, yet they are profoundly divergent in their clinical trajectories, systemic involvement, and therapeutic implications. While decades of molecular research have elucidated the roles of dystrophin, lamin A/C, DUX4, and sarcoglycans in maintaining muscle integrity [24,32,48,62], recent advances show that molecular diagnosis alone is insufficient for optimal patient management. Rather, the integration of genetic data with functional orthopedic phenotyping is now essential—not only for accurate diagnosis (e.g., early rigid spine pointing to *LMNA* mutations [17,19]), but for guiding timely, genotype-stratified interventions that preserve quality-of-life and prolong survival [9,11,21].

This review has deliberately reframed classical pathomechanisms—from sarcolemmal fragility to nuclear envelope dysfunction—through the lens of biomechanical consequence: how each defect translates into contractures, scoliosis, or gait deterioration long before overt weakness appears. In Duchenne muscular dystrophy, for instance, equinus contracture is not a late complication but an early biomechanical predictor of gait breakdown [20], and spinal fusion is no longer dictated solely by Cobb angle, but by a composite of pulmonary, nutritional, and cardiac status—with forced vital capacity ≥ 30–35% serving as a validated threshold for perioperative safety and long-term survival benefit [9,11]. Similarly, in *LMNA*-associated disorders, orthopedic planning is inseparable from cardiac risk stratification: prophylactic ICD implantation often precedes tendon lengthening or spinal stabilization, reflecting the clinical reality that sudden arrhythmic death may occur in patients with minimal motor impairment [12,13].

The emergence of disease-modifying therapies—from microdystrophin gene transfer to DUX4-silencing strategies—has extended life expectancy and redefined muscular dystrophies as chronic, multisystem conditions. Yet this success has exposed significant gaps: patients now survive into their fourth decade, but face novel, therapy-exacerbated phenotypes, including steroid-induced osteoporosis, atypical spinal rigidity, and obesity-related joint overload [21,42]. There remains no consensus on the guidelines for managing secondary deformities in this population—such as for adjacent segment disease after fusion, or heterotopic ossification post-release—nor for adapting rehabilitation to adults with decades of disuse atrophy superimposed on primary myopathy.

We argue that the next frontier lies in precision functional medicine—a framework where orthopedic surveillance intensity, imaging protocols, and surgical thresholds are explicitly tied to molecular diagnosis. For example, *LMNA* carriers warrant quarterly cardiac–orthopedic co-assessment from diagnosis; DMD patients on glucocorticoids require biannual bone density and spine imaging from age eight; and FSHD patients with very short D4Z4 arrays (1–3 repeats) merit early ophthalmologic and audiologic screening, alongside proactive functional monitoring [12,13,21,64,81]. Such stratification is no longer merely theoretical—it is feasible with current genetic tools and essential for equitable, effective care.

Ultimately, therapeutic success in 2030 should be defined not by dystrophin restoration alone, but by durable functional autonomy: self-toiletting at age 25, independent wheelchair-to-bed transfer at 35, or pain-free overhead function in FSHD. Achieving this vision demands deeper integration of neurology, genetics, and orthopedics—not as parallel disciplines, but as a single, unified framework guiding patients from molecular diagnosis to meaningful daily life.

## Figures and Tables

**Table 1:** Classification of limb-girdle muscular dystrophy (LGMD).

Subtype	Key Orthopedic Clinical Features	Critical Management Considerations
DMD (Dystrophinopathy)	Progressive muscle weakness, pseudohypertrophy, Gowers’ sign, early loss of ambulation [10,24].	Early spinal stabilization (Cobb angle > 20°) [9,70], management of steroid-induced osteoporosis, strict FVC monitoring [9,10].
EDMD (Emery–Dreifuss)	Early “triad” of contractures (Achilles, elbows, neck), rigid spine, early-onset cardiomyopathy [19,29].	Preoperative cardiac clearance (arrhythmia/SCD risk), early surgical release of contractures to maintain function [17,28]
LGMDR1 (CAPN3)	Thigh atrophy > hamstrings Scapular winging Myalgia	Pain is prominent—often underrecognized; stretching must avoid overstretching fragile fibers [71,72]
LGMDR2 (DYSF)	Distal onset (Miyoshi: gastrocnemius) Myoglobinuria	Eccentric exercise triggers rhabdomyolysis—contraindicated in rehab [73]
Sarcoglycanopathies (LGMDR3–6)	Rapid progression Early loss of ambulation Cardiac involvement in SGCB/SGCD (up to 50%) [32,74]	Cardiac screening must begin at diagnosis—cardiomyopathy may precede weakness [32]
Glycosylation defects (LGMDR7–13)	Broad spectrum: LGMD to congenital MD; CNS involvement in severe forms (lissencephaly) [75]	Respiratory insufficiency may be disproportionate to limb weakness—early spirometry crucial [76]
LGMDD1 (LMNA)	Late-onset axial/proximal weakness Severe cardiomyopathy and conduction defects Rigid spine [12,77]	See EDMD section—identical molecular pathology; cardiac risk dominates orthopedic planning [12]
LGMDD2 (DNAJB6)	Distal-predominant Asymmetric Low CK Mimics ALS	Misdiagnosis standard—early genetic testing prevents inappropriate interventions [77]

## Data Availability

No new data were created or analyzed in this study. Data sharing is not applicable to this article.
